# Annual alveolar bone loss in subjects with cardiovascular disease adjusting for associated systemic diseases and risk factors: a retrospective study

**DOI:** 10.1186/s12903-020-1015-y

**Published:** 2020-01-30

**Authors:** Mohammad Helmi, J. Max Goodson, Hatice Hasturk, Zuhair S. Natto

**Affiliations:** 1 Periodontics and Community Dentistry Department, College of Dentistry, King Saud University, Riyadh, Saudi Arabia; 2 Department of Oral Medicine, Infection, and Immunity, School of Dental Medicine, Harvard University, Boston, MA USA; 3 Department of Applied Oral Sciences, Center for Clinical and Translational Research, The Forsyth Institute, Cambridge, MA USA; 4 Department of Dental Public Health, Faculty of Dentistry, King Abdulaziz University, P.O.BOX 40311, Jeddah, 21499 Saudi Arabia

**Keywords:** Cardiovascular disease, Systemic diseases, Periodontal disease, Annual bone loss, Detect

## Abstract

**Background:**

To detect annual alveolar bone loss in subjects with cardiovascular disease (CVD) adjusting for associated systemic diseases and risk factors.

**Methods:**

A total number of 132 subjects that reported having CVD from 2008 to 2015 (*N* = 132). For longitudinal data analysis, 58 subjects eligible for inclusion with at least two exposures of complete mouth set or repeated BW radiographs with at least one-year interval compared with a control group. Alveolar bone level on mesial and distal sites of posterior teeth was measured on bitewing (BW) radiographs available in the electronic health records of each subject.

**Results:**

Subjects who reported having cardiovascular diseases experienced higher annual mean alveolar bone loss (0.062 mm per year) compared to Subjects with no cardiovascular diseases (0.022 mm per year).

**Conclusion:**

Subjects who have reported CVD had higher rate of annual bone loss compared to subjects who did not have any CVD. This observation indicates that targeting high-risk individuals for risk assessment is fundamental to provide the best healthcare possible to those who are the most in need. Periodic examination and assessment of periodontal health is an essential key factor for better oral health, however, it has to be more emphasized and prioritized for individuals that are more prone to the disease.

## Introduction

Many studies have been conducted to address the relationship between periodontal diseases and cardiovascular diseases [1–7] (CVD). In 2008, Humphrey et al. published a systematic review and meta-analysis based on seven cohort studies that revealed statistically significant association between periodontitis and the incidence of coronary heart disease [4]. Authors of the study concluded that the summary relative risk estimates for different categories of periodontal diseases (including gingivitis, periodontitis, bone loss, and tooth loss), to develop coronary heart disease, ranged from 1.24 to 1.34 (95% CI: 1.01–1.63). Moreover, DeStefano et al., found that individuals with more progressive periodontitis had 25% higher risk of developing coronary heart disease compared to individuals that had less progression of periodontitis [8]. Several studies have been conducted as well not to just assess the association or relationship between the two diseases, but also to investigate and understand the underlying inflammatory responses shared by periodontal diseases and cardiovascular diseases [1, 5–8].

The study of the link between periodontal diseases and cardiovascular diseases is not recent. A cohort study on men was conducted using joined data from the Normative Aging Study and the Dental Longitudinal Study between 1968 and 1971 [2]. The study hypothesized that periodontitis and coronary heart disease share same predisposing factors that might put individuals at higher risk of developing both of the diseases.

For general populations, several studies reported annual mean alveolar bone change or loss. In 1986, Albandar et al. published a 2-year longitudinal study that was conducted on 180 subjects that did not receive any periodontal procedures or treatments. Mean alveolar bone level was measured using radiographs over the two-year period and found that the total amount of bone loss detected for the whole population was 0.11 mm [9]. Moreover, studies on the natural progression of periodontal diseases in general populations, either clinical or radiographic, have estimated a mean annual clinical and radiographic bone loss equals to 0.05 mm [10, 11]. Another study, by Onabolu et al., estimated a radiographic mean alveolar bone loss of 0.2 mm – 0.3 mm per year after following 858 proximal sites over 6 years [12].

In a more recent systematic review and meta-analysis on the progression of periodontitis in terms of clinical attachment loss, radiographic bone loss, and tooth loss, Needleman et al. found that in general populations, including both full and partial mouth examination techniques, the mean annual attachment level change is 0.1 mm (95% CI 0.068, 0.13) [13, 14]. Although the aforementioned systematic review presented additional subgroup analyses of the effects of geographic location, gender, and age, the authors did not examine the effect of systematic diseases on the rate of mean annual bone level change/loss. Thus the aim of our study was to address this gap of knowledge comparing individuals who reported having cardiovascular diseases compared to individuals who are free of cardiovascular disease adjusting for associated systemic diseases and risk factors.

## Methods

The sample of this retrospective cohort study was obtained as a subpopulation from a previously selected sample for the estimation of prevalence of periodontitis [15, 16]. Information were gathered from an electronic health records system (AxiUm®) at Harvard School of Dental Medicine (HSDM), including several variables. The socioeconomic status (SES) was estimated using ZIP codes of all subjects and U.S. Census Bureau statistics which was previously explained [15, 16] (U.S. Census Bureau 2016). The subjects’ pool was selected based on their age at their last appointment at HSDM. One examiner (MH) reviewed all subjects records and, after implementing the exclusion criteria (described below), selected 1131 subjects that are suitable for analysis. We then, for the aim of this study, identified subjects that reported having CVD from 2008 to 2015 (*N* = 132). We examined the electronic health records of each subject to identify suitable radiographs for analysis.

### Exclusion methodology

Excluded subjects were previously explained [15, 16]. Briefly, less than 18 years old, no or unclear BW radiographs, absence of at least 2 approximating teeth, closed electronic files, and inability to do calibration with the measuring tool. For longitudinal data analysis, we required that eligible subjects for inclusion to have at least two exposures of complete mouth survey (CMS) radiographs or repeated BW radiographs with at least one-year interval. We identified 58 subjects that satisfied these criteria. This group is the exposure group; subjects who reported having CVD. 100 subjects of control group were also randomly sampled from the main sample (*N* = 1131) with the condition that everyone included being free of CVD. After examining each subject’s electronic health records and applying same exclusion methodology, a total of 87 subjects were identified and their BW radiographs were suitable for examination and analysis. For eligible subjects, teeth were excluded if it had certain criteria which were previously explained [15, 16].

**Fig. 1:**
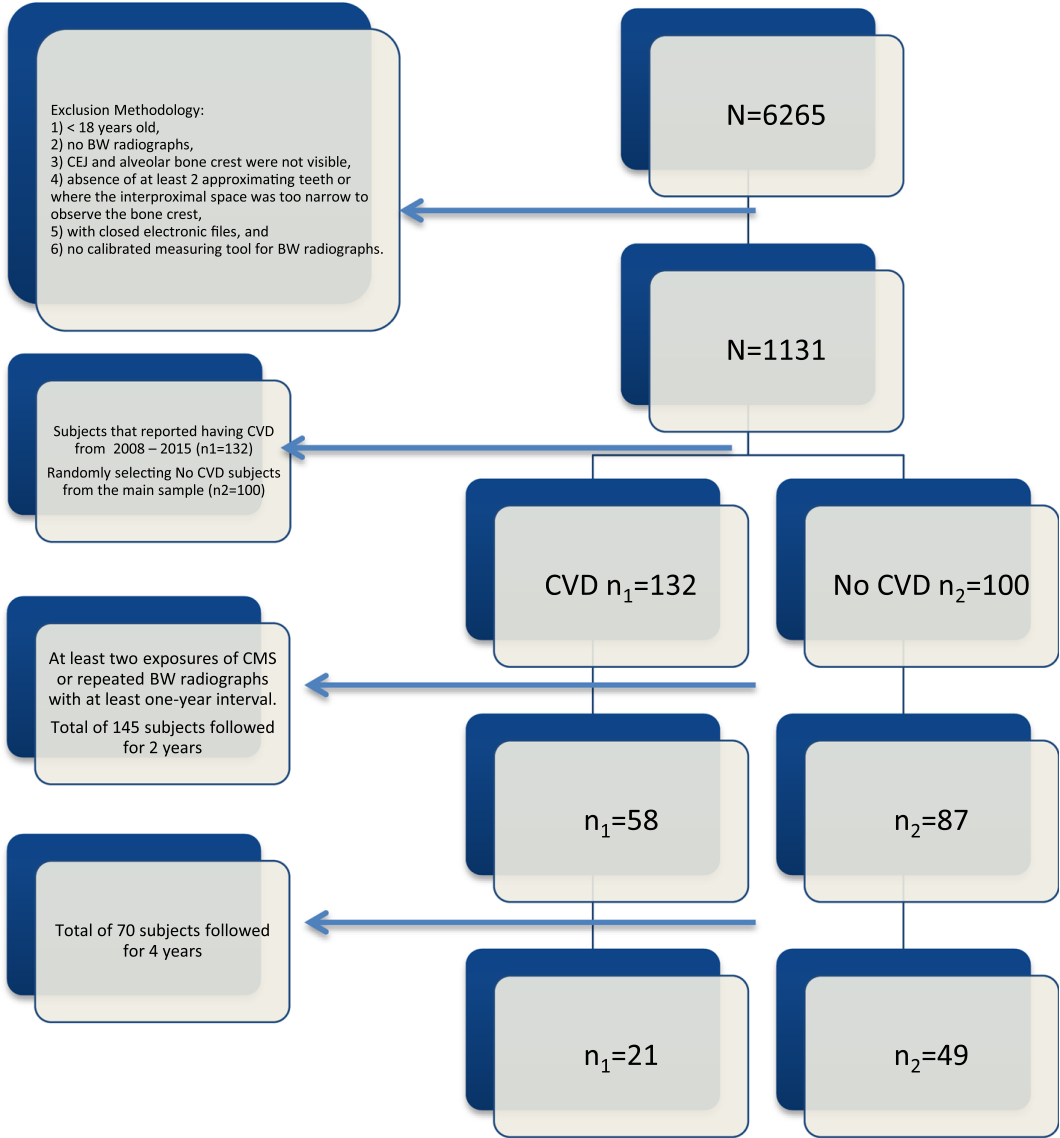
Flow chart of the exclusion methodology

Radiographs of the whole sample of 145 subjects (58 reported having CVD, 87 without CVD) were identified suitable for analysis over a two-year period. The sample has decreased after four-year period to a total of 70 subjects (21 with CVD, 49 without CVD) due to a lack of suitable radiographs for analysis. Flow chart of the exclusion methodology is presented in Fig. 1.

### Primary predictor

The primary predictor was whether the subjects had cardiovascular diseases (CVD) or not. Other variables were included in the model to adjust for any type of confounding expected. These variables included age, sex, race, BMI, median household income, smoking status, diabetes, and hypertension.

Data were gathered from the electronic health records of all subjects. Five categories of age were generated. Age groups of this study were defined as less than 30, 30–34, 35–49, 50–64, and 65 or more years old based on distribution of patients. BMI was measured based on the reported height and weight following Centers of Disease Control and Prevention criteria. (CDC 2017) Since household income was not included in the patient database, we based this variable on the ZIP code for each patient and the associated estimates of household income that have been collected using U.S. Census Bureau, 2012–2016 American Community Survey 5-Year Estimates.(U.S. Census Bureau 2016) The variable was categorized into either higher than the sample median household income (=1) OR equal or lower than the sample median household income (reference = 0). Also due to small numbers in each category, as well as the lack of information on frequency of cigarette smoking per day, we created a binary smoking variable for analysis by coding everyone who have ever smoked as ever smoker (=1) and those who had never smoked as never smoker (=0). To account for subjects who had higher risk or active disease of periodontal tissues, we included in the model procedure code D434 that is used by the clinicians at the school to indicate performing a procedure of scaling and root planning for 4 teeth or more for their subjects. Other codes provided in Table 1 were checked for frequencies and after including them in the analysis model, we found that D4341 is the only one that had a statistically significant influence on the outcome of interest and was included in the final model of analysis.

**Table 1:** Proportion of subjects received periodontal procedures including scaling and root planing comparing CVD and no CVD groups

N(%)			
Code	Description	CVD = 1	CVD = 0
D4240	Gingival flap for four teeth or more	0 (0)	0 (0)
D4241	Gingival flap for one to three teeth	1 (1.7)	0 (0)
D4260	Osseous surgery for four teeth or more	0 (0)	0 (0)
D4261	Osseous surgery for one to three teeth	3 (5.17)	0 (0)
D4263	Bone replacement graft	4 (6.9)	5 (5.7)
D4265	Biologic materials – tissue regeneration	2 (3.4)	4 (4.6)
D4266	Guided tissue regeneration	0 (0)	0 (0)
D4341	Scaling/root planing for 4 teeth or more	9 (15.5)	12 (13.8)
D4342	Scaling/root planing for 1–3 teeth	16 (27.6)	12 (13.8)
None		23 (39.6)	54 (62)
Total		58 (100)	87 (100)

*N* = 145 subjects

### Primary outcome

The primary outcome is the difference of mean alveolar bone level in millimeters between the group that were having CVD and the group that were free of any CVD, comparing mean bone levels at follow up visits to baseline mean of both groups which has been previously described [15–17, 18–22].

### Systemic diseases for control group

27 out of the 87 subjects in the control group had reported having diabetes, hypertension, or both. Table 2 presents frequency of systemic diseases over the CVD and no CVD groups. No other diseases were reported in the control group. We conducted two analyses, one with all 87-control subjects and one restricted to 60 individuals who were free of all diseases. The two analyses did not differ in terms of significance (data not shown).

**Table 2:** Systemic diseases distribution between the two groups

Distribution of systemic diseases among CVD group N(%)						
Group	Only CVD	CVD + Diabetes	CVD + Hypertension	C + D + H^a^	Free of all	Total
CVD	19 (32.7)	1 (1.7)	31 (53.5)	7 (12.1)	0 (0)	58 (100)
Distribution of systemic diseases among control group N(%)						
	CVD	Diabetes	Hypertension	D + H^b^	Free of all	Total
Control	0 (0)	13 (15)	13 (15)	1 (1)	60 (69)	87(100)

*N* = 145 subjects

^a^Subjects that reported having CVD, diabetes, and hypertension

^b^Subjects that reported having diabetes and hypertension

### Power calculation

Based on the main sample mentioned earlier (*N* = 1131), mean alveolar bone level was estimated to be 1.26 mm (±0.8). To detect at least 0.30 mm difference of mean alveolar bone level between subjects with CVD and subjects with no history of CVD, with α set to 0.05, we have 80% power to measure a real difference.

IRB approval [Protocol # IRB16–1838] was obtained through The Office of Human Research Administration, Harvard Faculty of Medicine. The study met the criteria for exemption per regulations found at 45 CFR 46.101(b) (4); “research involving the collection or study of existing data, documents, records, pathological specimens or diagnostic specimens, if these sources are publicly available or the information is recorded by the investigator in such a manner that the subjects cannot be identified directly or through identifiers linked to the subjects”, As such, additional IRB review is not required.

### Statistical analyses

Descriptive statistics (means and standard deviations for continuous variables, counts and percentages for categorical variables) were calculated. The percentage of subjects with periodontal bone loss and prevalence of periodontitis were computed for comparison between groups.

Mixed-effect linear regression model with multi-level design has been conducted to estimate the difference of change in mean bone level in mm comparing CVD group to no CVD group [17]. In this multi-level analysis, level 1 is the measured site level, level 2 is teeth level, and level 3 is participants’ level. Moreover, we included the time term to the model to assess the amount of change across the years of follow up. *P*-values less than 0.05 were considered statistically significant.

For examiner calibration and reliability as well as radiographic discrepancy adjustments, authors used the same methodology reported in their previous work (Prevalence of Periodontitis and Alveolar Bone Loss in a Patient Population at Harvard School of Dental Medicine – in-press).

## Results

60% of CVD group received periodontal treatments while 38% of no CVD received periodontal treatments Table 1. This also might indicate an increased risk of periodontal diseases for subjects with CVD as they are receiving more periodontal procedures compared to no CVD subjects.

### Descriptive statistics of baseline characteristics (Univariate analysis)

In descriptive statistics, the term bone level will be used as a description of the readings. A total of 145 subjects were included for analysis. Mean total alveolar bone level was 1.49 mm (±0.015). Mean age of the sample was almost 61-year-old (Ranged from 18 to 94) with 63% of the subjects being females (Table 3).

**Table 3:** Descriptive statistics and prevalence of mild, moderate, and severe periodontitis of the whole sample at baseline

Total	*N (%)*	% Mild PD	% Moderate PD	% Severe PD	MABL (mm)^a^	SE
	145 (100)	71.7	26.9	2.7	1.49	0.015
Age Groups (yrs)						
< 30	3 (2)	0.0	0.0	0.0	0.53	0.039
30–34	2 (1.4)	0.0	0.0	0.0	0.57	0.072
35–49	22 (15.2)	31.8	4.5	0.0	1.09	0.023
50–64	63 (43.4)	74.6	20.6	1.5	1.49	0.022
65+	55 (38)	90.9	45.4	5.4	1.81	0.027
Gender						
Male	53 (36.5)	67.9	24.5	1.8	1.42	0.026
Female	92 (63.5)	73.9	28.2	3.2	1.54	0.019
Race						
White	75 (51.7)	82.6	32.0	4.0	1.61	0.021
African American	9 (6.2)	77.8	22.3	0.0	1.42	0.059
Asian	7 (4.8)	85.7	42.8	0.0	1.71	0.087
Other	21 (14.5)	47.6	19.0	0.0	1.20	0.035
Unknown	33 (22.7)	64.7	17.6	0.0	1.38	0.030
Median Household Income						
Low	57 (39.3)	70.1	36.8	3.5	1.53	0.027
High	88 (60.7)	72.7	20.4	2.3	1.47	0.018
Body Mass Index						
Underweight	2 (1.4)	100.0	50.0	0.0	1.86	0.116
Normal	35 (24.1)	71.4	34.2	5.7	1.57	0.031
Overweight	37 (25.5)	67.5	21.6	0.0	1.48	0.031
Obese	33 (22.7)	63.6	18.2	3.0	1.33	0.031
Not reported	38 (26.2)	81.5	31.6	2.6	1.56	0.031
Smoking Status						
Never smoker	75 (51.7)	64.0	16.0	1.3	1.32	0.019
Former smoker	16 (11)	87.5	56.2	6.2	1.97	0.078
Current Smoker	7 (4.8)	85.7	42.8	0.0	1.68	0.053
Not reported	47 (32.4)	76.6	32.0	4.2	1.60	0.027
Diabetes						
Yes	22 (15.2)	68.1	9.1	4.5	1.34	0.042
No	123 (84.8)	72.3	30.0	2.4	1.52	0.016
CVD						
Yes	58 (40)	70.6	20.6	3.4	1.45	0.024
No	87 (60)	72.4	31.0	2.3	1.52	0.020
Hypertension						
Yes	52 (35.9)	63.4	19.2	3.8	1.44	0.027
No	93 (64.1)	76.3	31.1	2.1	1.52	0.018

^a^Mean alveolar bone level in millimeters

**Fig. 2:**
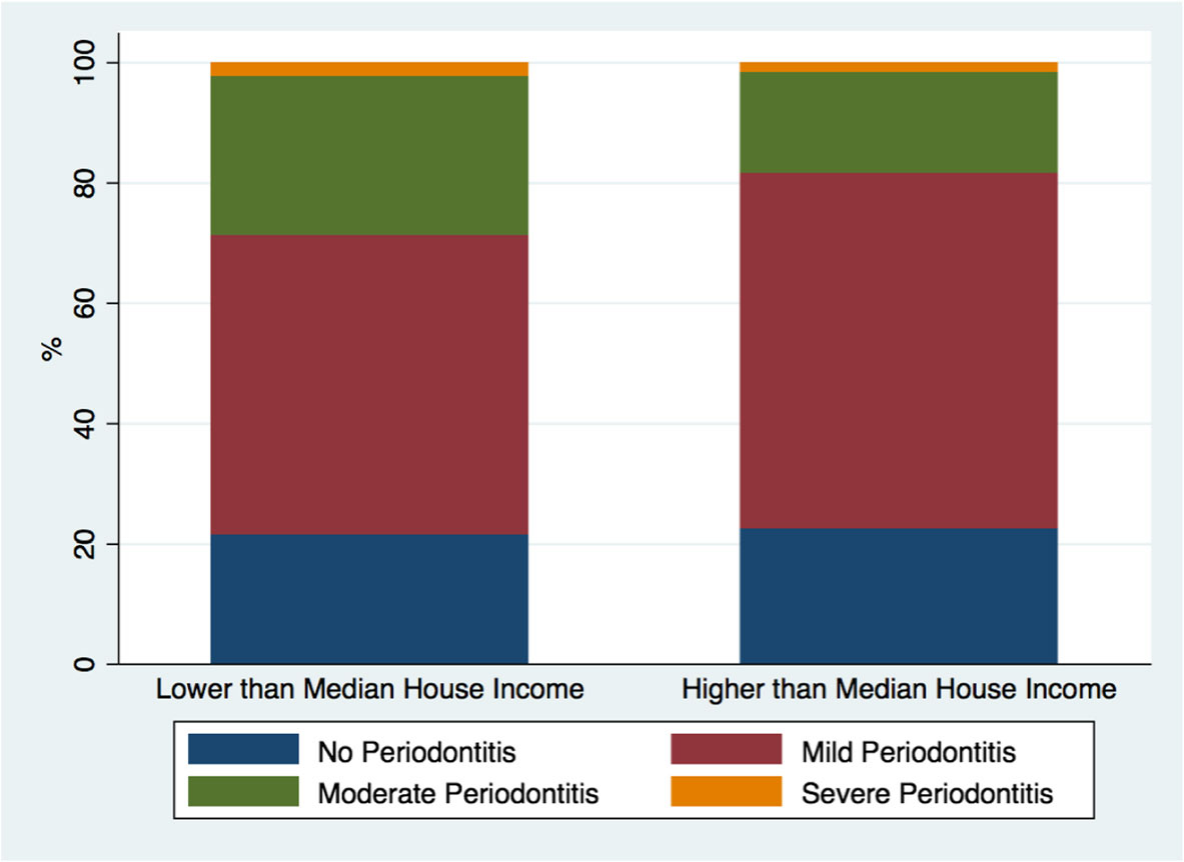
Prevalence of mild, moderate, and severe periodontitis by median household income

Overall mild periodontitis prevalence for the sample was 71.7% while moderate periodontitis prevalence was almost 27%. Severe periodontitis was the least prevalent by an estimate of 2.7% (±1.3) for the whole sample (Table 3). Moderate and severe periodontitis were higher among individuals with lower than median household income (Fig. 2). Table 4 presents selected variables comparing CVD to No CVD groups at baseline.

**Table 4:** Baseline Characteristics Comparing CVD group to No CVD group

Baseline Characteristics Comparing CVD group to No CVD group			
Variable	Overall	Subjects with CDV	Subjects with No CDV
Mean Age (sd)	60.9 (±12.7)	64.8 (±12.6)	58.3 (±12)
Age Range	18–94	29–94	18–78
MABL in mm (se^a^)	1.49 (0.015)	1.45 (0.024)	1.52 (0.020)
N (%)			
Females	92 (63.4)	34 (58.6)	58 (66.7)
Moderate PD	39 (26.9)	12 (20.6)	27 (31)
Severe PD	4 (2.7)	2 (3.4)	2 (2.3)
Low Household Income	57 (39.3)	25 (43.1)	32 (36.8)
Ever Smoker	23 (15.8)	11 (19)	12 (13.8)
Total (%)	145 (100)	58 (40)	87 (60)

^a^SE was calculated for MABL since it was multilevel measurements

### Unadjusted estimates overtime (bivariate analysis)

The term bone loss will be used to describe the amount of change of bone level between the two groups in this bivariate and the following multi-variable analyses.

Our results indicated that over two-year period, the group without CVD had 0.044 mm more bone loss compared to baseline (95% CI: 0.014, 0.075. *P*-value = 0.004) that increased to 0.120 mm (95% CI: 0.081, 0.159. *P*-value < 0.001) after 4 years compared to baseline. On the other hand, the group with CVD had experienced higher bone loss on both occasions of follow up compared to the group without CVD. After two years, CVD group had 0.122 mm more bone loss (difference) compared to the group without CVD (95% CI: 0.072, 0.172. P-value < 0.001) and 0.130 mm (95% CI: 0.061, 0.200. *P*-value < 0.001) difference in bone loss after four years compared to the group without CVD. Table 5 presents the estimates at baseline and over time.

**Table 5:** Crude and adjusted mean alveolar bone loss (mm) for both groups over time

Variables	Adjusted MABL (mm)** 95% CI	*p*-value	Unadjusted MABL (mm)** 95% CI	
Year*CVD				
0 No CVD (reference)				
2 No CVD	0.045(0.014–0.075)	0.004	0.044(0.014,0.075)	0.004
4 No CVD	0.121(0.021–0.160)	< 0.001	0.120(0.081,0.159)	< 0.001
0 CVD+	−0.022(−0.187–0.141)	0.784	−0.010(−0.192,0.172)	0.911
2 CVD+	0.121(0.071–0.172)	< 0.001	0.122(0.072,0.172)	< 0.001
4 CVD+	0.131(0.060–0.199)	< 0.001	0.130(0.061,0.200)	< 0.001
Age Groups (yrs)				
< = 34 (reference)				
35–49	0.408(0.01–0.80)	0.044		
50–64	0.889(0.50–1.27)	< 0.001		
65+	1.161(0.76–1.56)	< 0.001		
Gender				
Female (reference)				
Male	0.026(−0.12–0.17)	0.720		
Race				
White (reference)				
African American	0.026(−0.261-0.314)	0.854		
Asian	0.129(−0.19–0.45)	0.429		
Other	−0.123(− 0.33–0.09)	0.263		
Unknown	−0.082(− 0.25–0.08)	0.348		
Median Household Income (before interaction)				
Low (reference)				
High	−0.157((− 0.305)-(− 0.009))	0.037		
Body Mass Index				
Underweight	0.026(−0.26–0.31)	0.854		
Normal (reference)				
Overweight	0.129(−0.19–0.45)	0.429		
Obese	−0.123(− 0.33–0.09)	0.263		
Smoking Status				
Never smoker (reference)				
Ever smoker	0.237(0.037–0.4371)	0.020		
Diabetes				
No (reference)				
Yes	−0.140(−0.35–0.07)	0.194		
Median Household Income*Hypertension				
Low Not Hypertensive (reference)				
Low Hypertensive	−0.126(− 0.36–0.11)	0.294		
High Not Hypertensive	−0.110(− 0.29–0.07)	0.244		
High Hypertensive	−0.361((− 0.58)-(− 0.13))	0.002		
Hypertension (before interaction)				
No (reference)				
Yes	−0.195((− 0.36)-(− 0.02))	0.024		
D4341***				
No (reference)				
Yes	0.283(0.07–0.49)	0.007		
Random effect				
Individuals level	0.13(0.10–0.17)	n/a		
Teeth level	0.12(0.10–0.13)	n/a		
Sites level	0.20(0.19–0.21)	n/a		

*N* = 145 subjects (6945 sites from 1923 teeth)

* Statistical interaction

**Mean alveolar bone loss in millimeter

*** Scaling and root planing for 4 teeth or more code

### Adjusted estimates overtime (multi-variable analysis)

Estimated difference in means did not change drastically after controlling for other variables. After the two-year interval, the group without CVD had 0.044 mm more bone loss compared to baseline (95% CI: 0.014, 0.075. *P*-value = 0.004) that increased to 0.121 mm (95% CI: 0.021, 0.160. *P*-value < 0.001) after 4 years compared to baseline, controlling for age, sex, race, household income, BMI, smoking status, diabetes, hypertension.

The group with CVD however, had experienced higher bone loss on both occasions of follow up compared to the group without CVD. Subjects with CVD had 0.121 mm more bone loss compared to the group without CVD (95% CI: 0.071, 0.172. P-value < 0.001) after two years and 0.130 mm (95% CI: 0.060, 0.199. P-value < 0.001) more bone loss after four years compared to the group without CVD, adjusting for all other variables included in the model.

**Fig. 3:**
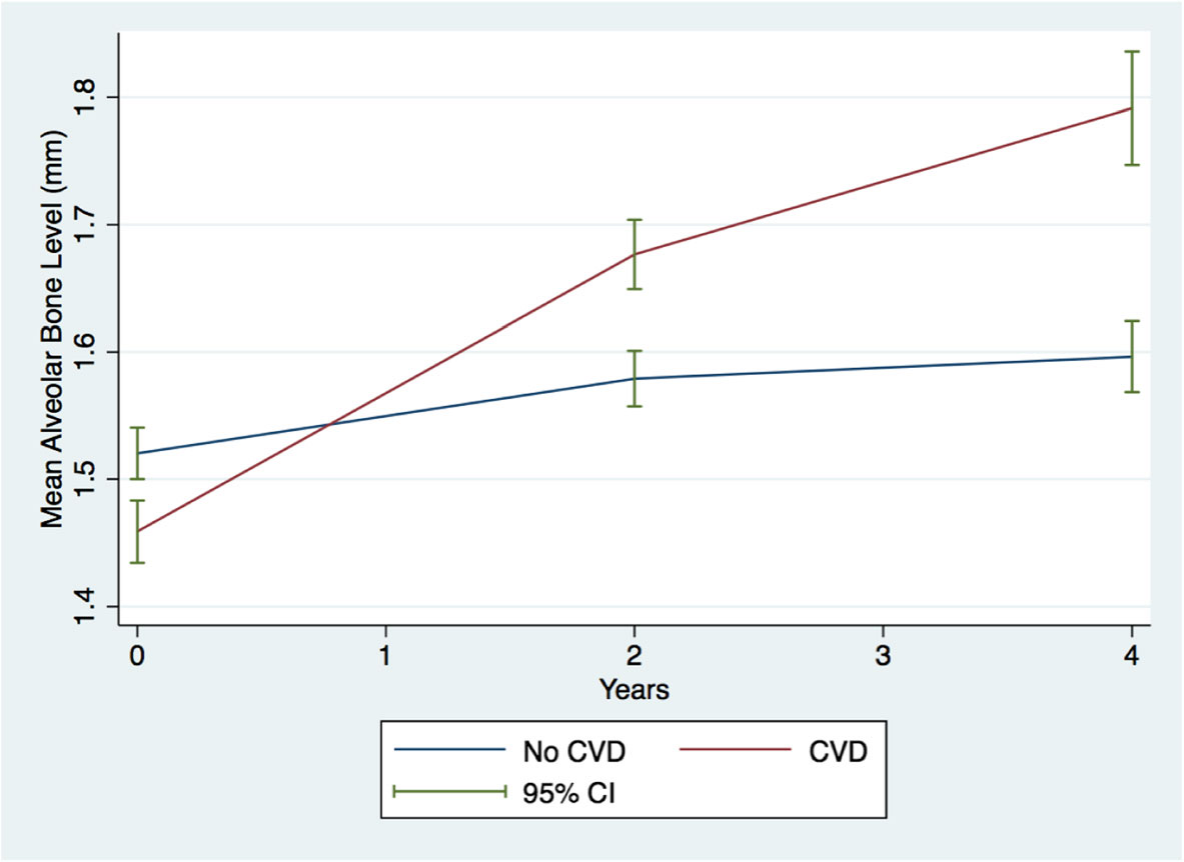
Mean alveolar bone level difference over time comparing CVD group to no CVD group

Table 5 presents the estimates at baseline and over time, in addition to the adjusted estimates of all other variables. The variables that were statistically significantly associated with our primary outcome (bone loss) were age, household income, smoking, and hypertension. Household income also showed a statistical significant interaction with hypertension with protective effect on bone loss. Figure 3 presents the change of bone loss comparing CVD group to no CVD group over the four-year period of time.

### Random-effect estimates

Comparing CVD group to no CVD group over time, the mean change or variability of alveolar bone level was 0.13 mm (95% CI: 0.10, 0.17) on the individual level, 0.12 mm (95% CI: 0.10, 0.13) on teeth level, and 0.20 (0.19–0.21) on sites level. Random-effect coefficients are also provided in Table 5.

## Discussion

Our results support that individuals with CVD have a higher risk of bone loss and periodontal diseases in general. Multiple studies found similar results and associations were observed between both diseases [2, 4, 8].

Furthermore, C-reactive protein (CRP), a protein that its level increases in acute inflammation, was also reported in literature to be associated with periodontitis and cardiovascular diseases that can put individuals at higher risk of developing the disease or to worsen the condition [23–25]. In 2003, moreover, Saito et al. found a statistically significant association between alveolar bone loss of posterior teeth and increased levels of CRP [26].

Another observation was reported in 2005 by Buhlin et al., after conducting a study to evaluate oral health of 143 age-matched women indicating that women with coronary heart disease had more pathological periodontal pockets, and vertical bone defects [3], compared to control group of women who did not have history of coronary heart diseases. They concluded that women with coronary heart disease had worse oral health in general compared to the control group [3].

Regardless of the increase in bone loss in the CVD group compared to no CVD group over time, our results also showed that at baseline the two groups did not have statistically significant difference comparing their mean alveolar bone levels. This can be a result of normal variation since the control group was randomly selected.

Although several studies in the literature reported an association between hypertension and periodontal diseases [27–29], we found that subjects with reported hypertension, who were living in areas where median household income was high, having lower bone loss compared to individuals who were living in areas where median household income was low. This is also supporting to the observation that individuals with high household income experienced lower difference in mean bone loss, which may indicate that access to healthcare system plays an important role by reducing the adverse effect of the outcome even among individuals who have predisposing conditions that put them at higher risk of the disease [30–35].

Nevertheless, limitations exist in this study. First, data were collected using partial mouth periodontal examination and therefore would result in underestimating the true rate of bone loss. Second, other risk factors influencing oral hygiene such as daily number of tooth brushing were not available in the records. We however accounted for subjects who underwent procedures involving scaling and root planning of 4 teeth or more based on the assumption that subjects receiving scaling and root planning of 4 teeth or more might indicate an active disease or an increased risk of developing the disease. Although we have accounted for all potential confounders available in our data, the relatively small sample size of the study may result in residual confounding that we could not account for. Moreover, estimating bone loss on radiographs with no clinical examination such as clinical attachment loss does not necessarily reflect active periodontal diseases but an indication of periodontal disease experience. This might result in underestimation of that time current experience of clinical signs of active periodontal disease.

### Clinical versus statistical significance

Although the clinical significance may appear small, the implications of this study emphasize the overall increased risk for individuals with CVD of having worsened periodontal health compared to individuals with no CVD. This small, though statistically significant, increase of annual alveolar bone loss of individuals with CVD compared to those with no CVD may result in much worse periodontal conditions over the years and define individuals with CVD as a high risk group. Special care and regular follow-ups are necessary to prevent much of negative outcomes for this vulnerable population.

## Conclusion

Subjects who have reported CVD had higher rate of annual bone loss compared to subjects who did not have any CVD. This observation indicates that targeting high-risk individuals for risk assessment is fundamental to provide the best healthcare possible to those who are the most in need. Periodic examination and assessment of periodontal health is an essential key factor for better oral health, however, it has to be more emphasized and prioritized for individuals that are more prone to the disease. The best quality of healthcare is fundamental right to all human beings. It is further more necessary to maintain best healthcare quality for individuals with conditions that put them at increased risk that might jeopardize their health such as cardiovascular diseases.

## Data Availability

The dataset used during the study are available from the corresponding author upon request.

## Abbreviations

AAP: American Academy of Periodontology
BMI: Body mass index
BW: Bitewing
CEJ: Cement-enamel junction
CMS: Complete Mouth Radiographic Series
CRP: C-reactive protein
CVD: Cardiovascular diseases
HSDM: Harvard School of Dental Medicine
IT: The information technology
SES: Socioeconomic status
